# YC-1 targeting of hypoxia-inducible factor-1α reduces RGC-5 cell viability and inhibits cell proliferation

**Published:** 2012-06-15

**Authors:** Leo Tsui, Tsorng-Harn Fong, I-Jong Wang

**Affiliations:** 1 Graduate Institute of Medical Sciences, College of Medicine, Taipei Medical University, Taipei, Taiwan; 2 Department of Anatomy, School of Medicine, College of Medicine, Taipei Medical University, Taipei, Taiwan; 3 Department of Ophthalmology, National Taiwan University Hospital, Taipei, Taiwan

## Abstract

**Purpose:**

The survival of retinal ganglion cells (RGCs) is a hallmark of many optic neurodegenerative diseases such as glaucoma. YC-1, a potential anticancer drug, is known to be able to decrease the stability and protein expression of hypoxia-inducible factor (HIF)-1α that is triggered by hypoxia and related to RGC survival. We hypothesized that YC-1 may alter RGC cell viability through the down-regulation of HIF-1α.

**Methods:**

Cell viability of the RGC-5 cell line was measured with a 3-(4,5-Dimethylthiazol-2-yl)-2,5-diphenyl tetrazolium bromide (MTT) assay. Flow cytometry, a LIVE/DEAD viability assay, and high-content screening (HCS) with MKI67 (K_i_-67) monoclonal antibodies were used to detect cell death and cellular proliferation.

**Results:**

We found that cells treated with 20 µM YC-1 for 24 h decreased the HIF-1α level in an RGC-5 cell line using immunoblotting and reduced the live cell number in an MTT assay. Results of flow cytometry and HCS demonstrated that reducing the cell proliferation of RGC-5 cells, not cell death, led to the decreased level in the MTT assay.

**Conclusions:**

Our findings demonstrate that YC-1-induced down-regulation of HIF-1α might reduce RGC cell proliferation and viability under normoxia, which implies a role of YC-1 in the neuroprotective effect for further clinical applications.

## Introduction

The retina is composed of seven main cell types, including retinal ganglion cells (RGCs), the only projection neurons that connect to the midbrain from photoreceptor cells [1]. RGCs can extend their axons through the optic nerve, the optic chiasm, and the optic tract into the superior colliculus and lateral geniculate nucleus, mainly on the contralateral side of the brain [1]. Loss of RGCs occurs in many ophthalmic conditions, such as glaucoma, diabetic optic neuropathy, etc., resulting from the process of cell apoptosis [2]. In animal models (monkeys and rabbits) of an axotomy and experimental glaucoma, it has been shown that RGCs possibly undergo apoptosis similar to the pathological changes that occur in glaucoma, and diabetic optic neuropathy [3-5], neurodegenerative diseases [6], anterior ischemic optic neuropathy, optic neuritis, optic nerve trauma, and AIDS [7].

There are several stimuli that may initiate apoptosis and result in the death of RGCs, such as neurotrophin deprivation, glial activation, excitotoxicity, ischemia, and oxidative stress [8]. These stimuli can also be triggered by an elevated intraocular pressure (IOP), which results in the release of neurotoxic factors, such as nitric oxide and tumor necrosis factor-α from retinal cells [9], a pressure-induced distortion of the lamina cribrosa leading to shearing and compressive forces on the RGC axons [10], or compression of the capillaries supplying the optic nerve head [11,12]. In these situations, hypoxia-inducible factor (HIF)-1α can be induced and expressed in RGCs to counter these stresses [2,13].

HIF-1α is a component of the transcription factor, HIF-1, and is triggered by hypoxic conditions [14]. The effects of HIF-1α on the expressions of many downstream genes, especially those involved in cell-cycle control and cell proliferation and death, are well established [15,16]. Moreover, HIF-1α stabilizers, such as cobalt chloride (CoCl_2_), are able to mimic hypoxia and are used in RGC programmed cell death models [17-20]. They are also able to induce the expression of β-amyloid precursor protein (APP) in RGCs as well as hypoxia [21], and they specifically upregulate Hsp27 after retinal ischemic preconditioning and prevent retinal ischemic damage both in vitro (RGC-5 cell line) and in vivo (rat retina) through HIF-1α activation [22]. These studies suggest that HIF-1α may play a key role in preventing hypoxia-induced RGC injury.

YC-1 (3-(50-Hydroxymethyl-20-furyl)-1-benzyl indazole) is a chemically synthetic benzyl indazole that directly activates soluble guanylate cyclase (sGC) to elevate cyclic (c)GMP levels in rabbit platelets and possesses antiplatelet activity [23]. Recently, it was found to be able to suppress HIF-1α expression in Hep3B cells and was suggested as a novel HIF-1α inhibitor [24]. Therefore, YC-1 is expected to become the first antiangiogenic anticancer agent to target HIF-1α, as it was found to halt tumor growth in immunodeficient mice grafted with five types of human tumor cells [25]. Yeo et al. [15] suggested YC-1 as a good lead compound for developing novel antiangiogenic and anticancer agents.

Due to the importance of HIF-1α in RGC programmed cell death and its downstream regulation of cell survival, we hypothesize that YC-1 might reduce HIF-1α expression and affect RGC cell viability. We first investigated the effect of YC-1 on HIF-1α protein expression in the RGC-5 cell line under normoxia. Then, cell viability, cell death, apoptosis, and proliferation were explored.

## Methods

### Cell culture

The RGC-5 cell line was purchased from American Type Culture Collection (Manassas, VA). Cells were cultured in medium composed of Dulbecco’s modified Eagle’s medium (Invitrogen Life Technologies, Carlsbad, CA) containing 4.5 g/l D-glucose, 2.5 mM L-glutamine, 110 mg/l sodium pyruvate, 100 U/ml penicillin/streptomycin (Invitrogen Life Technologies), 0.125 mg/l amphotericin B (Invitrogen Life Technologies), and 5% heat-inactivated fetal calf serum (Invitrogen Life Technologies) in a humidified incubator with 5% CO_2_ at 37 °C. The passage of RGCs used TrypLE (Invitrogen Life Technologies) following removal of the enzymes after centrifugation at 112× g for 5 min. Cells were not used until they had reached 80% confluence in our experiments.

### 3-(4,5-Dimethylthiazol-2-yl)-2,5-diphenyl tetrazolium bromide (MTT) assay

In our experiment, cell viability was determined by an MTT assay (Sigma-Aldrich, St. Louis, MO). The powder was dissolved at 5 mg/ml into distilled H_2_O and sterilized through a 0.22-µm filter before use. RGC cells were seeded in a 96-well plate (5,000 cells/well, total 100 µl) overnight. Then, cells were treated with different concentrations of YC-1 (Sigma-Aldrich) in dimethyl sulfoxide (DMSO; Sigma-Aldrich). After treatment, 10 µl of the MTT stock solution was added to each well and incubated with 5% CO_2_ at 37 °C for 2 h. Finally, we removed the medium, added 200 µl DMSO to each well, and incubated at room temperature for 15 min. The absorbance was measured at 570 nm on a microplate reader. All experiments were performed in four wells and repeated three times.

### Imaging

Photographic images were captured by a Zeiss Axiovert 35 Inverted Fluorescence Microscope (Carl Zeiss, Oberkochen, Germany) equipped with an Imaging Source DBK 41AU02.AS digital camera (Stuttgart, Germany). Fluorescence microscopy used a Leica DM 2500 stereomicroscope (Wetzlar, Germany) equipped with a Leica DFC490 digital camera. After treatment, cells were incubated with a LIVE/DEAD^®^ Viability/Cytotoxicity Kit (calcein AM/ethidium homodimer-1; Molecular Probes, Eugene, OR) and Hoechst 33342 (Invitrogen Life Technologies) for 30 min at room temperature.

### Flow cytometry

Treated cells were re-suspended in TrypLE followed by removal of the enzymes by centrifugation (112× g for 5 min). After filtration with a 0.45-µm filter, we counted the number of cells and diluted the suspension to 5×10^6^ cells/ml. Then, cells were incubated with a LIVE/DEAD^®^ Viability/Cytotoxicity Kit for 30 min or annexin V conjugated to allophycocyanin (APC; Invitrogen Life Technologies)/propidium iodide (PI; Sigma-Aldrich) for 15 min at room temperature. Finally, cells were analyzed by BD LSR II and FACSCalibur flow cytometry (Becton Dickinson, San Jose, CA).

### High-content screening (HCS)

We seeded RGC-5 cells in a Costar^®^ 96-well black solid plate (5,000 cells/well, total 100 µl; Bio-Rad Laboratories, Hercules, CA) using a BioTek MicroFill Microplate Dispenser (BioTek Instruments, Winooski, VT). After treatment, cells were fixed with 4% paraformaldehyde in phosphate-buffered saline (PBS, pH 7.4; Invitrogen Life Technologies) for 15 min and allowed to stand with 0.2% Triton-X in PBS for 15 min. After washing with PBS twice, cells were blocked with 5% BSA (BSA) in PBS for 1 h, followed by incubation with the NCL-L-Ki67-MM1 primary antibody (1:200; Novocastra Lab, Newcastle upon Tyne, UK) for 1.5 h and the secondary antibody, Alexa Fluor^®^ 488 goat anti-mouse immunoglobulin G (IgG; 1:200; Invitrogen Life Technologies), for 1 h. Finally, our sample was analyzed using a Thermo Scientific Cellomics^®^ ArrayScan^®^ VTI HCS Reader (Thermo Fisher Scientific, Pittsburgh, PA), and the imaging output used the software Columbus™ Image Data Storage and Analysis System (PerkinElmer, Columbus, OH).

### Western blotting

Cells were washed with PBS twice, and the total protein was extracted using ice-cold lysis buffer composed of 10% RIPA (Sigma-Aldrich) and a proteinase inhibitor cocktail (Sigma-Aldrich). After centrifugation at 18,994× g and 4 °C for 30 min, the protein concentration was determined using an R-250 Protein Assay kit (Bio-Rad Laboratories, Philadelphia, PA). Samples were mixed with 5× sample buffer (312.5 mM Tris-base, 50% glycerol, 12.5% β-mercaptoethanol, 10% sodium dodecyl sulfate [SDS], and 0.01% bromophenol blue) and dry-heated to 95 °C for 5 min before SDS–PAGE (PAGE) with an 8% acrylamide gel. The separated proteins were transferred onto polyvinylidene difluoride (PVDF) membranes and blocked with PBS containing 5% non-fat milk overnight. The membranes were incubated with the primary antibodies, an anti-rabbit HIF-1α antibody (1:1000; Epitomics, Burlingame, CA) and anti-mouse-β-actin antibody (1:10^4^; Millipore, Bedford, MA), at room temperature for 2 h. After washing with TBST (25 mM Tris, 150 mM NaCl, 2 mM KCl, and 0.1% Tween-20; pH 7.4) for 5 min three times, the membranes were incubated with an anti-rabbit or anti-mouse horseradish peroxidase-conjugated secondary antibody (1:10^4^; Santa Cruz Biotechnology, Santa Cruz, CA) at room temperature for 1 h and washed with TBST three times. Finally, immunoblotting was visualized using an enhanced chemiluminescence detection kit (Millipore).

### Statistical analysis

Our data are presented as the mean±standard deviation (SD). The statistical significance of differences between groups was determined using Student’s *t*-test. A p value of <0.05 indicated a statistically significant difference.

## Results

### Effect of YC-1 on HIF-1α expression in RGC-5 cells

**Figure 1:**
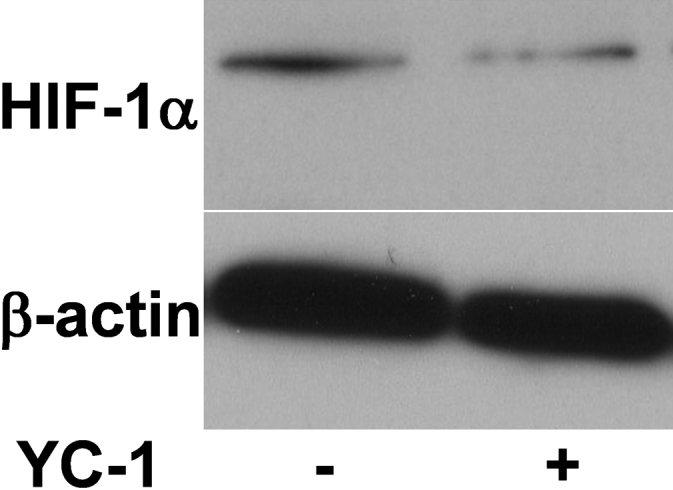
YC-1 decreased hypoxia-inducible factor (HIF)-1α protein expression in RGC-5 cells. Total proteins were extracted from RGC-5 cells treated with 20 µM YC-1 for 4 h. Expressions of HIF-1α and β-actin were determined by western blotting. β-actin was used as the internal control.

The effects of YC-1 on the protein synthesis and stability of HIF-1α are well established in vivo and in vitro [24,26-28]. Here, in RGC-5 cells, we also demonstrated that YC-1 similarly decreased HIF-1α expression by western blotting (Figure 1).

### Effect of YC-1 on RGC-5 cell viability

**Figure 2:**
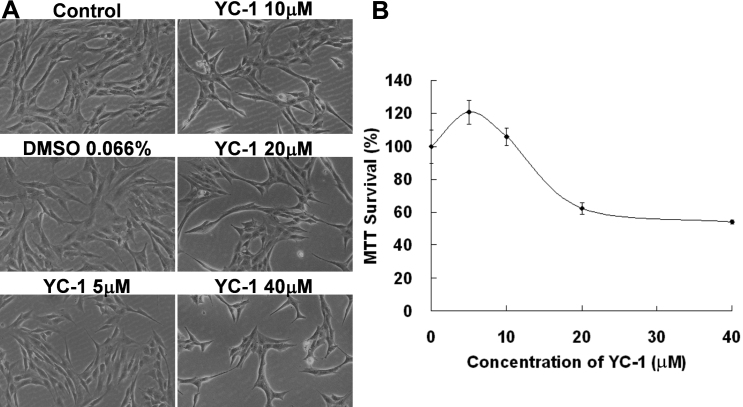
YC-1 induced dose-dependent retinal ganglion cell (RGC) cell death. **A**: RGC-5 cells were exposed to 5, 10, 20, and 40 µM YC-1 and 0.066% DMSO for 24 h. Morphological changes in cell density were observed with light microscopy. **B**: Cell viability was detected with an MTT assay of increasing the YC-1 concentration to treat RGC-5 cells. * Indicates p<0.05 compared to the DMSO vehicle group.

**Figure 3:**
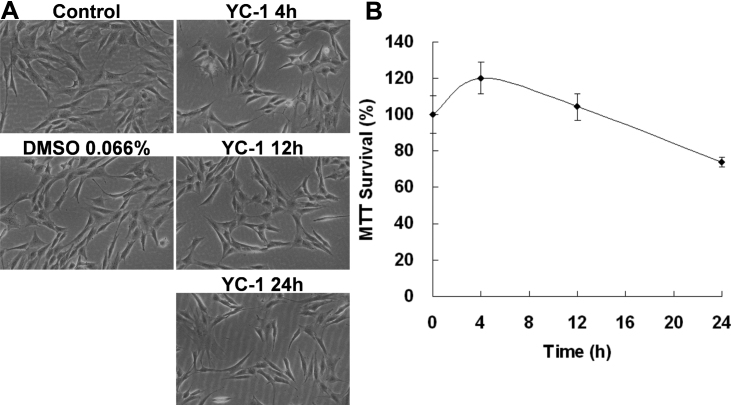
An increase of the YC-1 exposure time resulted in a decrease of the RGC-5 cell density. **A**: RGC-5 cells were exposed to 20 µM YC-1 for 4, 12, and 24 h. Morphological changes in cell density were observed with light microscopy. **B**: Cell viability was detected by an MTT assay of RGC-5 cells treated with YC-1 for different incubation times. * Indicates p<0.05 compared to the DMSO vehicle group.

MTT is a formazan dye that is reduced enzymatically by involvement with NADH or NADPH in live cells [29,30]. An MTT assay is commonly used to estimate cell survival, growth, and differentiation in response to extracellular activators or toxic agents [31]. We used an MTT assay to measure changes in the number of live RGC-5 cells after being exposed to YC-1. Our results revealed that the absorbance at 570 nm in the MTT assay displayed a dose- and time-dependent response after RGC-5 cells were exposed to YC-1 (Figure 2B and Figure 3B). Exposure to low concentrations of YC-1 did not induce RGC-5 death until >20 µM (Figure 2B). Cells treated with 20 µM YC-1 for 24 h exhibited ~30% reduced cell viability compared to the control group. Short-term incubation (4 and 12 h) of RGC-5 with 20 µM YC-1 had no effect on the MTT assay and even resulted in an insignificant increase in the cell number (Figure 3B). Morphological results also showed that YC-1 indeed decreased the cell density of RGC-5 cells (Figure 2A and Figure 3A). These findings established that YC-1 actually reduced the number of live cells.

### Effect of YC-1 on the number of apoptotic RGC-5 cells

**Figure 4:**
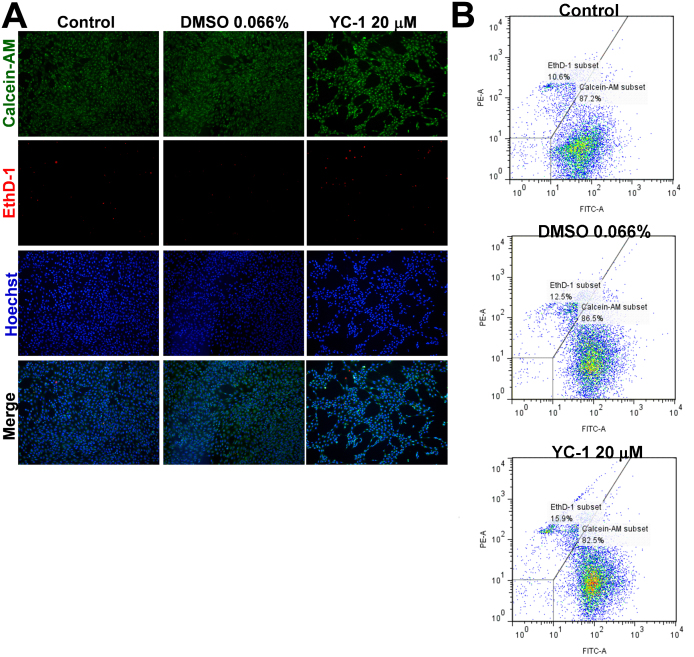
YC-1 had no influence on RGC-5 cell death. **A**: After treatment with 20 µM YC-1 or 0.066% DMSO for 24 h, RGC-5 cells were stained with calcein AM (green for live cells), EthD-1 (red for dead cells), and Hoechst 33342 (blue for nuclei) for 40 min at room temperature. Representative photomicrographs show the cell density and composition. **B**: Cell viability was examined by calcein AM/EthD-1 fluorescence staining and flow cytometry.

The MTT assay is widely used in cytotoxicity and cell proliferation screening. We hypothesized two possible reasons for the decreased absorbance of MTT after YC-1 exposure: induction of cytotoxicity and inhibition of cell proliferation. We first analyzed the cytotoxicity of YC-1 using calcein AM and EthD-1 dyes, respectively, to determine the number of live and dead cells. The non-fluorescent calcein AM is known to be cell permeable, and it converts to the intensely fluorescent calcein by intracellular esterase in live cells and then emits green fluorescence. When EthD-1 binds nucleic acids, it undergoes a 40-fold enhancement in fluorescence. However, EthD-1 can be excluded by the intact plasma membranes of live cells, but it presents a bright-red fluorescence in dead cells. Our findings demonstrated that YC-1 decreased the density of live cells but did not significantly increase the density of dead cells compared to the control group (Figure 4A), as was also found by flow cytometry (Figure 4B). This indicates that YC-1 should be a noncytotoxic agent toward RGCs.

**Figure 5:**
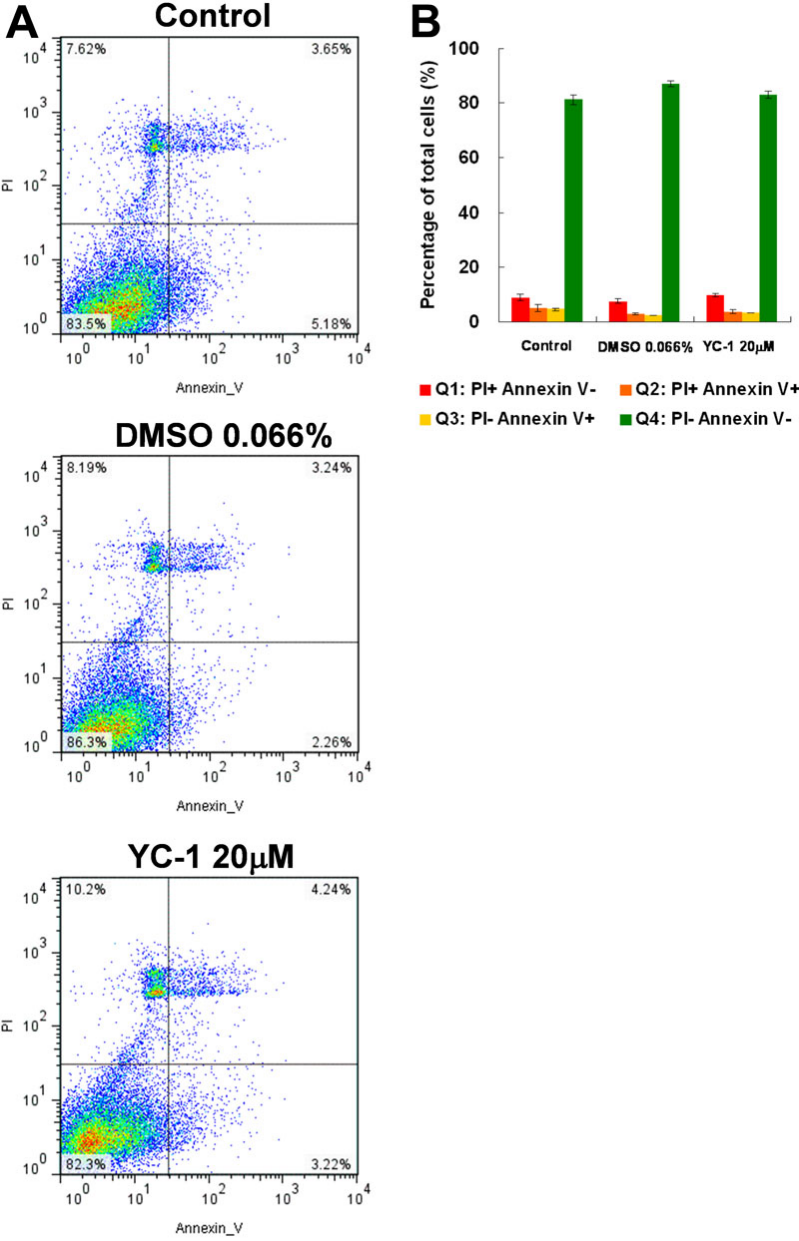
There was no significant increase of apoptotic RGC-5 cells after treatment with YC-1. **A**: RGC-5 cells were treated with 20 µM YC-1 or 0.066% DMSO for 24 h. Apoptosis of cells was assessed using annexin V/propidium iodide staining and flow cytometry. **B**: The histogram shows the representative flow cytometry data of three experiments.

To confirm these findings, we further used annexin V and a fluorescent nucleic acid dye, PI, with flow cytometry to differentiate apoptotic and necrotic cells. Annexin V is a calcium-dependent protein probe that detects an early apoptotic marker, phosphatidylserine, on cell surfaces [32]. Our data indicated that YC-1 did not significantly change the ratio of apoptotic and necrotic cells (Figures 5A,B). Taken together, we determined that YC-1 suppressed RGC-5 survival but did not induce programmed cell death. Accordingly, we inferred that YC-1 may decrease the number of live cells by inhibiting cell proliferation and consequently result in the decreased absorbance of MTT.

### Effect of YC-1 on RGC-5 cell proliferation

**Figure 6:**
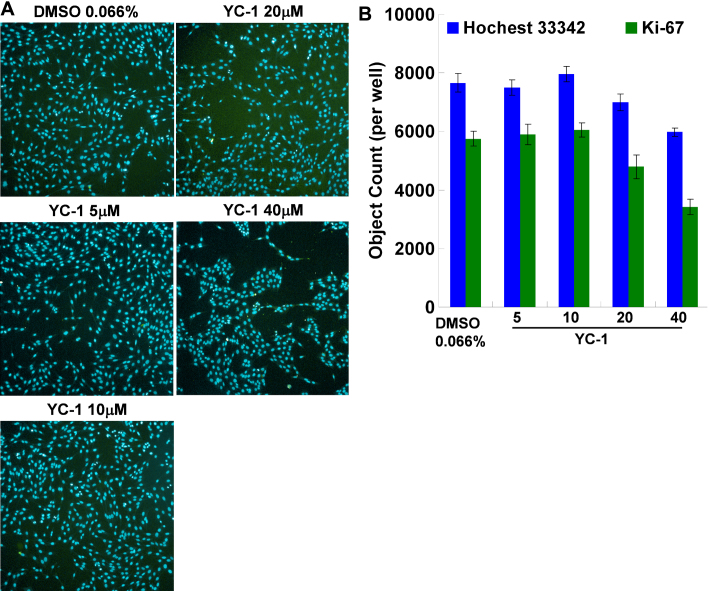
YC-1 reduced RGC-5 cell proliferation. **A**: YC-1 decreased the number of nuclei and the K_i_-67 cell proliferation marker expression in RGC-5 cells using Cellomics^®^ high-content screening and the Columbus™ system. RGC-5 cells were treated with 5, 10, 20, and 40 µM YC-1 or 0.066% DMSO for 24 h. After fixation and immunofluorescence staining, cells were detected and analyzed by an HCS reader. Photos show fluorescence staining results of Hoechst 33342 (blue) and K_i_-67 (green). **B**: The quantification results of HCS. * Indicates p<0.05 compared to the Hoechst-stained nuclei of the DMSO vehicle group. ^#^ Indicates p<0.05 compared to the K_i_-67-stained cells of the DMSO vehicle group.

K_i_-67 is a nuclear protein and a proliferation marker that exists in all periods of the cell cycle except the resting stage (G_0_) [33]. To further clarify the role of YC-1 in cell proliferation, we used HCS to detect nuclear K_i_-67 expression after YC-1 treatment. HCS is an efficient technique that is combined with numerous hardware improvements and quantitative measurements of cellular imaging to collect data from complex biologic systems [34]. Results showed that YC-1 markedly reduced the density of MKI67 (K_i_-67) expression (Figure 6A). K_i_-67 expression in the nuclei of cells treated with 20 µM YC-1 for 24 h decreased ~15% compared to that of the control group (Figure 6B). Our findings confirmed that YC-1-induced RGC-5 loss was independent of cell death and apoptosis but was related to the decrease in the number of live cells and inhibition of cell proliferation.

## Discussion

The present study explored the inhibitory effect of YC-1 targeting HIF-1α on RGC-5 survival under normoxia. We found that YC-1 directly attenuated HIF-1α expression and subsequently decreased the number of live cells, according to an MTT assay. However, YC-1 significantly lowered RGC-5 cell viability but had little to no influence on dead and apoptotic cells using flow cytometric measurements of calcein AM/EthD-1 and annexin V/PI fluorescent staining. The HCS results showed that the decrease in live cells was likely due to a reduction in cell proliferation. Our results indicated that YC-1 should be noncytotoxic in vitro, although it interfered with RGC-5 survival.

HIF-1α was stabilized under hypoxia but rapidly degraded in normoxic conditions [35]. Many studies have mentioned the role of HIF-1α in RGCs under hypoxia, but few have discussed its role under normoxia. It is well established that YC-1 can act on the response domain of HIF-1α and promote HIF-1 degradation [26-28]. Our immunoblotting results showed that YC-1 attenuated the HIF-1α protein level in RGC-5 cells under normoxia. The findings suggest that HIF-1α is physiologically required to maintain RGC cell survival.

HIF-1 is a heterodimeric protein composed of HIF-1α and HIF-1β subunits. The HIF transcription system can act as a key regulator of responses to changes in oxygen levels and activates more than 70 genes that provide adaptation to ischemia and oxidative stress [35,36]. HIF-1 has also been mentioned as a potential medicinal target for neurodegenerative diseases, like Alzheimer, Parkinson, and Huntington’s diseases and amyotrophic lateral sclerosis [37]. In this article, we demonstrated a correlation between HIF-1α levels and RGC viability. We inferred that the neuroprotective role of HIF-1α may regulate downstream genes involved in RGC growth and proliferation under normoxia.

Many studies have examined the mechanisms of YC-1-inhibited cell proliferation. Tulis et al. suggested that YC-1 inhibited cell proliferation through a cGMP-dependent mechanism in rat vascular smooth muscle cells (VSMCs) [38]. In contrast, other studies have reported that the mechanism was cGMP-independent in human umbilical vein endothelial cells [39], primary rat thoracic aorta VSMCs [40], HA22T and Hep3B cells [41], and rat mesangial cells [42]. Furthermore, it has been reported that YC-1 induced S-phase arrest under hypoxia via an HIF-1α-related pathway in Hep3B, HEK293, and Caki-1 cells [43]. This finding corresponds with our hypothesis and provides evidence of HIF-1α-regulated RGC survival.

The loss of RGCs is an important characteristic of glaucoma and diabetic eye disease [3-5], which has been implicated by the results of hypoxia [2,44-46]. The vulnerability of RGC loss might result from its sensitivity to acute, transient, and even mildly systemic hypoxic stress [47]. In this study, we demonstrated that inhibition of HIF-1α protein expression results in a decrease of RGC viability. Our results indicated that HIF-1α could be a potential target able to control the progress of RGC survival-related eye disease and a crucial target for drug development in treatments of cancer, heart disease, and stroke [48].

As YC-1 is able to reduce RGC viability without inducing cell death, we think that YC-1 should be independent of RGC survival-related optic pathologies. Recently, Song et al. established that YC-1 can inhibit HIF-1 expression and laser-induced choroidal neovascularization in rats [49]. On the other hand, YC-1 was found to suppress pathological retinal neovascularization and enhance physiologic revascularization of the retinal vascular plexuses in a mouse with oxygen-induced retinopathy by impairing the ischemia-induced expression of HIF-1 and its downstream angiogenic molecules [50]. Moreover, YC-1 was shown to elevate the IOP in rabbits [51] and decrease the human trabecular meshwork cell volume [52]. Evidently, the definite effects of YC-1 on ocular development and progressive eye diseases need to be clarified.

Our findings demonstrate that YC-1 can reduce the viability of RGCs and inhibit cell proliferation. These results may cause down-regulation of the HIF-1α signal pathway. Nevertheless, HIF-1α has been reported to possess the essential ability to cause arrest of the cell cycle during hypoxia [53]. This suggests the possibility that YC-1 affects RGC cell proliferation in hypoxic conditions. The relationship between HIF-1α and RGC survival under normoxia and hypoxia should be further discussed in the future.
